# Whose Norms, Whose Prejudice? The Dynamics of Perceived Group Norms and Prejudice in New Secondary School Classes

**DOI:** 10.3389/fpsyg.2020.524547

**Published:** 2021-01-07

**Authors:** Luca Váradi, Ildikó Barna, Renáta Németh

**Affiliations:** ^1^Nationalism Studies Program, Central European University, Budapest, Hungary; ^2^Department of Minority Studies, Faculty of Social Sciences, Eötvös Loránd University, Budapest, Hungary; ^3^Department of Social Research Methodology, Faculty of Social Sciences, Eötvös Loránd University, Budapest, Hungary; ^4^Department of Statistics, Faculty of Social Sciences, Eötvös Loránd University, Budapest, Hungary

**Keywords:** prejudice, adolescence, perceived norms, Spiral of Silence, contact theory, pluralistic ignorance, Hungary, anti-gypsyism

## Abstract

Ethnic prejudice can lead to exclusion and hinder social integration. Prejudices are formed throughout socialization, and social norms inform individuals about the acceptability of prejudice against certain outgroups. Adolescence is a crucial period for the development of intergroup attitudes, and young people are especially prone to follow the norms they perceive in their reference groups. At the same time, the effect of perceived norms on prejudice in school classes has been rarely studied. In Hungary, where prejudice against the Roma is widespread and there is no clear social norm proscribing prejudiced manifestations, this topic is especially relevant. In the present paper, based on multi-level analyses of panel data from Hungarian ninth-graders, we find that adolescents adjust their attitudes to those they perceive to be dominant among their classmates and that classmates serve as more important reference groups than teachers do. More contact with Roma is found to be associated with less prejudice against them. Looking at school classes, we find that at the beginning of the school year, many students underestimate the rejection of prejudiced expressions in their classes. By the end of the year, many students are found to adjust their own attitudes to the falsely perceived class norm. Based on our findings, we argue that school classes should be treated as important normative contexts for the socialization of intergroup attitudes and should receive special attention from both scholars and practitioners working in the fields of prejudice research and reduction. Furthermore, we suggest that teachers can most successfully hinder prejudices by working on a common, visible, shared class norm rather than “teaching” students that prejudices are not acceptable.

## Prejudice in Adolescence

The development of prejudice in children has been researched extensively (e.g., Allport, 1954; Aboud, 1988, 2005; Aboud and Doyle, 1996; Baker and Fishbein, 1998; Nesdale, 1999, 2004, 2008; Nesdale and Flesser, 2001; Fishbein, 2002; Aboud and Amato, 2003; Nesdale et al., 2005; Levy and Killen, 2008), but the period of adolescence has received much less attention (Bar-Tal and Teichman, 2005; Schiefer et al., 2010; Raabe and Beelmann, 2011; Váradi, 2014; Miklikowska, 2018; Miklikowska et al., 2019a). Most of the theories on prejudice acquisition agree that attitudes toward outgroups are based on the (social) identity of the individual (e.g., Nesdale, 1999, 2004; Aboud, 2005; Killen et al., 2010), and adolescence is a crucial period for the formation of the identity (Erikson, 1959; Cole and Cole, 2001; Durkin, 2001; for an overview see Steinberg, 2008). This is the period when developing teenagers have to find an answer to such questions as “who am I?” or “what do I believe in?” (Shaffer, 2009). Hence, the age period of adolescence is of major importance in the development and crystallization of intergroup attitudes (Krosnick and Alwin, 1989; Alwin and Krosnick, 1991; Henry and Sears, 2009; Rekker et al., 2015; Bohman et al., 2019).

When looking at how children’s intergroup attitudes develop, Raabe and Beelmann (2011), in their meta-analysis of 113 research reports from all over the world, have found clear trends showing a peak in prejudice in middle childhood (5–7 years) followed by a slight decline in late childhood (8–10 years). No such trend, however, has been found among adolescents, whose attitudes also shift but without following a clear, general pattern. According to the authors, the influence of the social context on prejudice increases by the age of adolescence (Raabe and Beelmann, 2011).

Aboud (2005) in the Integrative Frame Model of Prejudice Development does not hypothesize clear directions of attitude development in the period of adolescence. Moreover, she argues that peers and social cues become gradually more important in the development of prejudice over time, especially in adolescence. Bar-Tal and Teichman (2005) make a similar argument in their Integrative Model of Formation of Stereotypes and Prejudice and emphasize the role of the social context and messages from the ingroup in addition to micro-level personality factors. When comparing children and adolescents, they also point to an increased influence of peers and the school context among adolescents. At the same time, authors of both of the above models call for further empirical studies among adolescents to better understand the role of peers and social norms in the formation of prejudice. In line with this, Mitchell (2019) also argues that in order to understand the effect of the social context on attitude formation, more studies should focus on adolescents.

## Prejudice and (Perceived) Social Norms

Allport devoted a full chapter of his classic 1954 book, *The Nature of Prejudice*, to conformity (Chapter 17) as he viewed social norms and conforming to them a key element in young people’s acquiring of prejudicial attitudes. Crandall and Stangor, in their 2005 review, argue that Allport’s thesis has not only been proven right but, in fact, conformity does play an even more important role in the spread of prejudice than previously thought.

In line with Sherif and Sherif’s (1953) Group Norm Theory, Crandall et al. (2002) found in a series of correlational studies that people closely follow the perceived norms when they express prejudice and also match their intended behavior to what they perceive as acceptable in their reference groups. Moreover, a number of experimental studies demonstrated that perceived social consensus guides individual attitudes related to stereotypic beliefs and behaviors (Haslam et al., 1996; Wittenbrink and Henly, 1996; Sechrist and Stangor, 2001; Stangor et al., 2001a,b).

Perception is a key element in the functioning of social norms, as social norms are not written down and accessible in most cases. This is the very reason, namely, that individuals usually do not have access to information describing the objective norm in their groups, why Tankard and Paluck (2016) argue that individuals base their subjective perceptions of norms on their local experiences. However, these “estimations” are rarely accurate and perfectly match the actual rates of acceptance of certain attitudes and behavior. Thus, there is evidence in the social psychology literature, pointing to the discrepancy between what people personally think and what they perceive others think about an issue.

Pluralistic ignorance occurs when people falsely estimate the majority attitude (Van Boven, 2000) and has been defined as “shared false ideas” by Shamir and Shamir (1997). The discrepancy between personal beliefs and perceptions of these has been studied for over 80 years, starting with Katz et al.’s (1931) study documenting the rejection of black members from university fraternities based on the false perception of other members’ segregationist views. Pluralistic ignorance has been found in relation to a wide range of individual attitudes and perceived majority attitudes, including the acceptance of racial segregation (O’Gorman, 1975, 1979) and prejudice (Bergmann, 1988; Váradi, 2014; for an overview see: Mendes et al., 2017). It can take the form of full misperception, when people believe that everyone else has the same opinion that is different from theirs, or, in less severe cases, people significantly over- or underestimate the public support of certain opinions and ideas (Shamir and Shamir, 1997). In relation to intergroup attitudes, the false perception of the majority view often follows a typical pattern: people individually report being more tolerant compared to when they were asked about how they perceived the attitudes of their social environment or the population at large. This special type of pluralistic ignorance, which is typical for the false overestimation of the acceptance of prejudice in society, has been described as “conservative bias” by Fields and Schuman in 1976 and occurs when people individually have more tolerant values than the perceived majority view in their own group (Fields and Schuman, 1976). Systematic bias has been found to occur almost exclusively in this specific, so-called conservative direction (Shamir and Shamir, 1997). The question occurs whether or not it might be the case that people are inclined to hide their “real views” in the interview situation to appear to be complying with the norm banning the expression of prejudice and, therefore, express their true opinions by projecting them to the whole population or group. This hypothesis has been tested early on in a series of experiments measuring pluralistic ignorance and behavioral outcomes and no supporting evidence was found (Fields and Schuman, 1976).

In Hungary, conservative bias has been found in relation to attitudes toward Roma in two correlational studies: adult respondents overestimated Hungarian people’s discriminatory intentions toward Roma (Angelusz, 2000), and teenagers overestimated the acceptability of anti-Roma remarks in their school classes (Váradi, 2014). Róbert Angelusz in his studies of pluralistic ignorance among the Hungarian adult population replicated the original study of Fields and Shuman focusing on attitudes toward Roma in Hungary during the 1990s. Participants were first asked to give their own opinion about a contact situation with a Roma child and then they were asked to estimate the answer of the majority of the people in Hungary. The survey was carried out in 1994 on a representative sample of the Hungarian population above age 14, *N* = 1000. The contact situation was about a little girl asking her mother whether she could invite her Roma playmate to their home. Answer possibilities were: “*1. the child should not be allowed to play with Gypsy children; 2. they can play together in the school but not at home; 3. she can invite her to their home.*” Angelusz found a significant discrepancy between the actual opinions and the estimated ones: while only 12% of the respondents assumed that people would allow the child to come to their house, in fact, 53% of the respondents chose this option. Angelusz found similar patterns when asking about how much people would accept Roma neighbors (Angelusz, 2000).

Later, focusing on adolescents, Luca Váradi also found a discrepancy between the perceived acceptance of anti-Roma remarks and the actual responses of her study participants (Váradi, 2014). The study was carried out in 2010 on a sample of 1038 students in 46 classes in Budapest. Respondents were aged between 12 and 19 years. In this case, participants were asked to imagine that a classmate made anti-Roma remarks and were asked to estimate the extent to which most of their classmates would agree with this. Then, they were asked to report how much they would personally agree. In this case, as full school classes were included in the study, comparison of the perceived and actual majority view was possible. Results were similar to those found by Angelusz: while only 32% of the respondents assumed that most of their classmates would not agree with a student making anti-Roma remarks, in fact 52% of the respondents went to a school class in which majority (more than 50%) of the students reported that they would not agree with this.

Such shared false perceptions might lead to the persistence or even to the increase of negativity toward outgroups. Based on the Spiral of Silence Theory (Noelle-Neumann, 1984) people who believe that the group norm supports their views are more confident in their communication, whereas those who believe to belong to the minority with their opinion tend to remain silent because of their fear of isolation from the group. In case of conservative bias, even if a majority of the group members have non-prejudiced attitudes, since they (falsely) feel to belong to an “attitude-minority,” the norm of the acceptance of prejudice might seem to be unquestioned because of the non-prejudiced group members’ silence. Thus, according to the Spiral of Silence Theory, the fear of isolation, coupled with conformity guiding group members to follow the perceived group norm, lead to an increased acceptance of prejudice by time.

As previous studies related to pluralistic ignorance in Hungary had a cross-sectional design, these dynamics have only been implied but not yet tested. School classes serve as ideal settings for the testing of the Spiral of Silence Theory for two reasons. First, pluralistic ignorance, i.e., the correctness of perceptions, can be directly tested by comparing the perceived acceptability of prejudice in the class to the actual attitudes of the students (Henson and Denker, 2007). Second, in the age of adolescence, both the fear of isolation and conforming to the peer group are central motivational processes (Zhang et al., 2016). Using panel data, the present study allows for the detection of attitude changes over time both at the individual and at the group level serving as a window through which the formation of new secondary school classes can be observed.

## Reference Groups in Adolescence

Conformity is especially prominent in the age of adolescence when teenagers need to follow the norms of several reference groups outside of their homes and have a high need for acceptance (Berndt, 1979; Knoll et al., 2015; Zhang et al., 2016). Social Identity Theory suggests that groups the individuals feel to belong to influence their attitudes (Tajfel, 1974; Hewstone et al., 2002; Smith and Louis, 2008; Roth et al., 2018). Applying the Dynamic Social Impact Theory (Nowak et al., 1990) to adolescents, it can be hypothesized that if a student identifies with his or her school class, the classmates’ attitudes and behavior should be an important source of information and the individual will follow them. Accordingly, by following the group norms, adolescents socially connect with their own group and this way avoid exclusion (Crandall et al., 2002; Paluck, 2011). Thus, classmates should serve as a significant reference group throughout adolescence (Brown, 2004; Thijs et al., 2016), and the perceived norms of the peer group play a role in the expression of prejudice and prejudiced behaviors of teenagers as they adjust to the current peer consensus or perceived norms (for an overview, see Sechrist and Stangor, 2005; Paluck, 2011; Hjerm et al., 2018). Testing the influence of peer leaders, trained to intervene against prejudiced behavior in high schools, Elizabeth Levy Paluck found that they were able to change the behavior of their close contacts among their classes but not the climate of the entire class (Paluck, 2011).

The school itself is a primary institution of socialization outside of the family with many different types of learning experiences for students. This is where students come into contact with authorities and learn about the consequences of (not) following the institutional rules. School experiences in adolescence were found to influence political views and engagement and general trust in institutions (for an overview, see Eckstein and Noack, 2015). While schools are embedded in different layers of contexts, including the political climate, the state curriculum, and the local realities, such contexts also exist within schools including the attitudes of teachers and the specific class climates. These different layers or analytical levels might all interact and have an effect on the attitudes of students (Gniewosz and Noack, 2008).

The school class itself can also be viewed as a socializing agent, where the aggregated attitudes of the individual’s peers are posited to influence their own (Mitchell, 2019). Classroom climates, consisting of distinct, shared group norms of the students have been found to exist among German (Gniewosz and Noack, 2008), Hungarian (Váradi, 2014), and Swedish (Miklikowska et al., 2019a) secondary school students and Swedish elementary school students (Mitchell, 2019) and to correlate with individual intergroup attitudes. Though some are only based on correlational evidence, these findings still point to the importance of school classes in the formation of intergroup attitudes. This is especially prominent in the case of Hungary where secondary school students spend most of their time together with their classmates for the 4 (or more) years in secondary education.

Students do not only spend lots of time among their classmates, but teachers are also important actors in their socialization (Grütter and Meyer, 2014; Miklikowska et al., 2019b). The role of teachers and student–teacher relationships in students’ intergroup attitudes has been investigated in some studies, and teachers’ support and trust toward their students have been found to hinder students’ intergroup prejudice in multi-ethnic settings in Western Europe (Grütter and Meyer, 2014; Geerlings et al., 2017; Miklikowska et al., 2019b). To our knowledge, however, the effect of perceived teacher norms on the formation of prejudice in adolescence has not yet been studied in Central and Eastern Europe. Nevertheless, this question should not stand by itself, but in relation to the effect of perceived norms among classmates. The reason for this is that adolescents might perceive the norms related to the acceptability of prejudice among teachers and classmates to be different. It is important to know the (perceived) norms of which reference group adolescents follow.

## Intergroup Contact

As entering a new school and thus a new social environment also means that students have a chance to come into contact with people belonging to various groups, it is important to know how these encounters might affect their intergroup attitudes.

The Contact Hypothesis (Allport, 1954) assumes that direct contact with outgroup members may reduce prejudice and foster positive intergroup attitudes if the contact occurs under certain conditions: it is a cooperative, institutionally supported contact situation between individuals of equal status who pursue common goals (for an overview, see Pettigrew et al., 2007; Pettigrew and Tropp, 2013). Allport’s original conditions for “optimal contact” have been further tested and developed by Pettigrew and Tropp, including friendship as an ideal type of contact for prejudice reduction (Pettigrew, 1998; Pettigrew and Tropp, 2006; Pettigrew et al., 2007). Contact has been found to reduce not only prejudice toward members of the group the contact person belongs to but also, through its secondary transfer effect, prejudice toward further outgroups (Schmid et al., 2012). Furthermore, contact has been found to prove effective in reducing prejudice indirectly, by knowing that an ingroup friend has an outgroup friend (Paolini et al., 2007; Pettigrew et al., 2007).

Even though all the above examples, and especially Pettigrew and Tropp’s impressive meta-analysis (Pettigrew and Tropp, 2006), point to a correlation between more contact and less prejudice, the question of causality arises. Namely, whether it is the contact that reduces prejudice, or, on the contrary, does prejudice reduce contact making prejudiced individuals less willing to engage with members of outgroups? Though longitudinal evidence is less rich compared to the varsity of correlational studies, in a study of three Western European countries, both directions have been found to be significant, though the direction of contact reducing prejudice was found to be much stronger than the opposite effect (Binder et al., 2009), whereas another longitudinal study among British students only found the effect of contact predicting prejudice to be significant (Brown et al., 2007).

## Context—Prejudice Against the Roma in Hungary

Roma people are Europe’s largest ethnic minority group, currently numbering 10–12 million people who have been systematically excluded and marginalized at institutional and state levels in many European countries (European Union Agency for Fundamental Rights, 2019). Though there is a lack of research about the level of prejudice against the Roma in Europe, it is clear from the few existing studies that anti-Roma prejudice is widespread and severe (Zick et al., 2011; Loveland and Popescu, 2016; Kende et al., 2017). In Hungary, Roma constitute the largest ethnic minority group, accounting for 6–10% of the country’s population. Prejudice against the Roma is common in Hungary with representative studies indicating that a high percentage of the adult population agree with negative stereotypes, would not prefer social contact with Roma people, and support segregation (European Union Agency for Fundamental Rights, 2018; Orosz et al., 2018). As Kende et al. (2017) argue, anti-Roma prejudice can be seen as the norm in Hungary.

Though there have been fewer studies about the younger generations’ attitudes toward the Roma, the existing ones suggest similar patterns to what has been found among adults (for an overview, see Váradi, 2014). Váradi (2014) argues that as long as the social norms accepting anti-Roma prejudice do not change, there is little chance that young people in Hungary will grow up to become less prejudiced than their parents.

Though Roma form the largest ethnic minority group in Hungary, they are hardly present in the school curriculum, and according to recent studies investigating the content of textbooks, if yes, this is mostly not in a way that would question the existing negative stereotypes (for a detailed Hungarian language overview, see Bogdán, 2016; Council of Europe, 2020). Without representative studies among teachers, we do not know their attitudes toward Roma, though a recent experimental study including pre-service teachers found prejudice and discriminatory tendencies against Roma students (Bruneau et al., 2020). Furthermore, as school segregation is common, especially on the secondary level, contact opportunities are limited (European Commission, 2018; Radó, 2019). In this sense, it is questionable to what extent education in Hungary is able to reduce prejudice.

## The Present Study

The first aim of the present study was to investigate individual-level correlates of anti-Roma prejudice and the change thereof during the first year of secondary school among Hungarian teenagers. Second, at the level of school classes, we aimed to investigate the relationship between the perceived class norm related to the acceptance of anti-Roma remarks at the beginning of the year and the actual acceptance of such remarks at the end of the year. For this, we used panel data collected at two time points during the first year of secondary school among Hungarian adolescents, measuring their intergroup attitudes and perceptions of related norms.

In line with the literature on perceived social norms, conformity, and prejudice (Crandall and Stangor, 2005; Sechrist and Stangor, 2005; Tankard and Paluck, 2016), as a first set of hypotheses, we predicted substantial links between perceived norms and prejudice. More specifically, we expected that adolescents’ anti-Roma prejudice would be correlated with the perceived acceptance of anti-Roma remarks among teachers (Hypothesis 1a) and classmates (Hypothesis 1b) and that the correlation with perceived acceptance among teachers would be mediated through that among classmates (Hypothesis 1c). In relation to this, we also predicted (Hypothesis 1d) that the change in the perception of the acceptance of prejudice would be coupled with a change in prejudice itself.

As a second hypothesis, in line with the literature on intergroup contact (Pettigrew et al., 2011; Orosz et al., 2016), we predicted a substantial link between the diversity of the social network and prejudice. We expected that more diversity of the social network of the individual will be correlated with less prejudice against Roma (Hypothesis 2a). We also predicted that an increase in the diversity of the social network will be associated with a decline in the level of anti-Roma prejudice from the beginning to the end of the school year (Hypothesis 2b). We furthermore predicted (Hypothesis 2c) that, due to the secondary transfer effect of contact (Schmid et al., 2012), not only more Roma contacts but also more contacts from other outgroups will be associated with less prejudice. Based on the findings of previous studies on indirect contact (Paolini et al., 2007; Pettigrew et al., 2007), we predicted that (Hypothesis 2d) direct contact with Roma will be more strongly associated with prejudice than indirect contact. Finally, we predicted that indirect contact with Roma will be negatively correlated with anti-Roma prejudice, even after controlling for direct contact with Roma (Hypothesis 2e).

Relying on earlier findings in the field of pluralistic ignorance regarding the perception of intergroup attitudes (Angelusz, 2000; Váradi, 2014), as a third set of hypotheses, we predicted (Hypothesis 3a) that a substantial proportion of our participants would underestimate the level of rejection of anti-Roma remarks among their classmates at the beginning of the school year. Consequently, in line with the Theory of the Spiral of Silence (Noelle-Neumann, 1984) and literature on conformity (Crandall and Stangor, 2005), we predicted (Hypothesis 3b) that classroom climate (majority view) in classes in which conservative bias is present would adjust to the misperceived class norm and would become more accepting of anti-Roma remarks by the end of the school year.

## Design and Procedure

We analyzed data from a larger panel survey, titled “class climate, attitude climate.” The research aimed at scrutinizing the formation of group norms and intergroup attitudes in Hungarian secondary school classes, measuring students’ attitudes at two time points, at the beginning and at the end of the first year of secondary school, which is the ninth grade.

Principals and teachers from the participating schools were informed about the study, and consent forms were sent to the parents to decide about their child’s participation. The questionnaires were filled out in schools during class time in the computer labs. A member of the research team was always present to ensure the quality of the data collection. Prior to opening the link to the questionnaire, participants were informed about the nature of the study, anonymity, and voluntary participation.^1^ The first round of data collection took place upon the beginning of the school year in September and October; the second round took place 8 months later, in the final weeks of the school year.

The study was designed in a way to minimize social desirability pressure as students were not responding to questions from an interviewer but were sitting in front of personal computers. The presence of the member of the research team ensured that teachers do not intervene and that the respondents do not communicate with each other during data collection.

### Participants

Participants of the study were in the ninth grade, starting their first year of secondary school in newly formed classes. The sample was a proportionally stratified probability sample of secondary schools in the Hungarian capital city, Budapest, representing the heterogeneity of school types according to academic track (vocational and grammar school) and maintaining body (state or church). A total of 1400 students in 59 classes from 32 schools filled out the first round of the questionnaire, and 1100 students filled out the second round, out of which 896 participants filled out both questionnaires.

For our regression models, we selected those respondents who participated in both waves and had valid values for all variables in the models. This resulted in 891 respondents from 49 classes. In this subsample, students were 15 years of age on average (SD = 0.57) during the first wave of data collection. Among the respondents, 53% were female and all of them belonged to the majority ethnic group, Hungarian^2^.

### Measures

Intergroup attitudes of Hungarian teenagers were measured by an online questionnaire using measures from the *Attitudes and Friendships* study (Váradi, 2014) and some newly developed measures based on a qualitative pre-study. In order to ensure that the items are understood correctly by adolescents and that they measure what they are intended to, the questionnaire has been pretested through a series of cognitive interviews (Beatty and Willis, 2007).

#### Anti-Roma Prejudice

In our analysis, we use variables measuring two different aspects of anti-Roma prejudice. The first aspect grasps the respondents’ level of prejudice against the Roma, while the second grasps their perception of their classmates’ and teachers’ attitudes toward the expression of anti-Roma prejudice.

To measure the respondent’s level of anti-Roma prejudice, we created two indices for both time points. As factor weights were very similar, we decided to calculate the mean values of the variables instead of using principal component analysis:

1. How would you feel about having a Roma student as your desk-mate? (0—would be unhappy; 10—would be happy).
2. Would you accept a friend of Roma origin? (0—would not accept; 10—would accept and be happy).
3. How would you feel about having Roma classmates? (0—would be unhappy; 10—would be happy).
4. How would you feel about having Roma schoolmates? (0—would be unhappy; 10—would be happy).
5. How do you feel about the Roma? (0—I do not like them, 10—I like them).

We used Cronbach’s alpha to assess the internal consistency of the items. The values of Cronbach’s alpha are 0.95 and 0.96 for the two measurements, which suggest excellent reliability. The minimum value of the created composite measure is 0, meaning that the respondent has no anti-Roma prejudice, while the maximum value of 10 suggests the opposite^3^.

#### Perception of the Acceptability of the Expression of Anti-Roma Prejudice

To measure the respondents’ perception of the acceptability of anti-Roma expressions among their classmates and teachers, we asked them what they thought most of their classmates and teachers would think if a classmate made anti-Roma remarks. Variables ranged from 0 to 10, where the lowest value meant that the respondent thinks that most of the classmates/teachers would not agree, while the highest value that the majority of the given group would agree with a student making anti-Roma remarks. We also asked the respondents about their own reactions. They answered using a similar 11-point scale where 0 meant that the respondent would not agree with the classmate’s anti-Roma remark, while 10 meant that he/she would^4^.

#### Network Diversity (Self-Reported)

Based on the theories described above, we used network diversity as an independent variable. We asked about the number of friends and acquaintances the respondents have from certain ethnic or religious groups. These outgroups have been identified based on the qualitative pre-study, having selected groups that were mentioned in the discussions as clearly defined outgroups. Moreover, we inquired about extended contact by asking about the number of the respondents’ friends who have friends from these groups. The questions were the following:

1. How many friends do you have from these groups? [Hungarians from Transylvania; Roma; Chinese; Jews; foreigners from a different country] (0; 1; 2; 3; more than 3).
2. How many acquaintances do you have from these groups? [Hungarians from Transylvania; Roma; Chinese; Jews; foreigners from a different country] (0; 1; 2; 3; more than 3).
3. How many friends do you have who have one or more friends from any of these groups? [Hungarians from Transylvania; Roma; Chinese; Jews; foreigners from a different country] (0; 1; 2; 3; more than 3).

To measure the diversity of the respondent’s network, we created an index for each time point by summing these variables. We used Cronbach’s alpha to assess the internal consistency of the items. The values suggest excellent reliability as they are 0.85 and 0.88. The minimum value of the created composite measure is 0, meaning that the respondent’s network is not diverse at all, while those having the maximum score of 60 have an extremely diverse network. In order to have a clear understanding of the effects of Roma contacts and contacts from other outgroups, a separate index measuring the number of Roma contacts has been created. We created an overall index including all variables concerning Roma contacts. For further analysis, we separated direct and indirect Roma contacts. For direct contacts, we created an index of the number of Roma friends and acquaintances of the respondent, while for indirect contact, we used the number of friends who have Roma friends.

#### Measures of Change Between the Two Measurement Points

When analyzing the determinants of change in anti-Roma prejudice, we regressed change in prejudice on predictors, a regression model referred to as “gain score modeling” in the literature (see, e.g., Gelman and Hill, 2007, pp. 177–178). We created the dependent variable by subtracting the prejudice at the second time point from that at the first time point. The positive values of the new variable thus mean that the respondent’s prejudice increased, zero means that it did not change, and the negative values can be interpreted as a decrease. Similarly, for independent variables, such as the perception of the acceptability of anti-Roma prejudice among teachers and classmates, as well as network diversity, we defined its change between the two waves by subtraction.

## Results

### Descriptive Statistics

Descriptive statistics for all variables used in the analyses are depicted in Table 1. Participants of our study were found to follow the general patterns of prejudice among Hungarian adults and adolescents, reporting widespread prejudice against the Roma at both time points (mean value in the first time point: 5.67 and in the second: 5.39). Although there was no substantial change between the beginning and the end of the school year, prejudice against the Roma became somewhat lower (paired samples *t*-test *p* < 0.001, Cohen’s *d* = 0.120). However, it is interesting that not only the respondents would agree more with a classmate’s anti-Roma remark (paired samples *t*-test *p* < 0.001, Cohen’s *d* = −0.123), but the respondents think the same about their teachers (paired samples *t*-test *p* < 0.001, Cohen’s *d* = −0.177) and classmates (paired samples *t*-test *p* < 0.01, Cohen’s *d* = −0.090). The biggest change can be seen in the respondents’ network diversity, as for the second measurement, it became much more diverse (paired samples *t*-test *p* < 0.001, Cohen’s *d* = −0.393).

**TABLE 1:** Descriptive statistics of the variables.

**Continuous variables**	***M***	**SD**	**Minimum**	**Maximum**	**No. of items**	**Cronbach**’**s alpha**	***n***
W1 Anti-Roma prejudice	5.67	2.94	0	10	5	0.95	891
W1 Acceptability of anti-Roma prejudice by the respondent	3.17	3.05	0	10	1	−	891
W1 Perception: teachers	2.09	2.30	0	10	1	−	891
W1 Perception: classmates	4.07	3.03	0	10	1	−	891
W1 Network diversity	17.80	13.27	0	60	5	0.85	891
W2: Anti-Roma prejudice	5.39	2.88	0	10	5	0.96	891
W2 Acceptability of anti-Roma prejudice by the respondent	3.52	3.07	0	10	1	−	891
W2 Perception: teachers	2.60	2.54	0	10	1	−	891
W2 Perception: classmates	4.37	3.00	0	10	1	−	891
W2: Network diversity	22.14	14.37	0	60	5	0.88	891
**Categorical variable**	**Categories**						
Gender	0: male						415
	1: female						476

*Source: Class climate, attitude climate study. The authors’ own computation. Perception—teachers: Perception of the acceptability of anti-Roma prejudice among the respondents’ teachers. Perception—classmates: Perception of the acceptability of anti-Roma prejudice among the respondents’ classmates.*

Table 2 shows the correlation between the measures under research. As can be seen from the table all independent variables have significant correlation with the variable measuring anti-Roma prejudice in both waves. Perceived norms among teachers and classmates were, as predicted in Hypotheses 1a and 1b, found to be correlated with anti-Roma prejudice at both time points, the perceived norm among classmates being a much stronger correlate of anti-Roma prejudice than that of teachers.

**TABLE 2:** Pearson’s correlation between the variables.

		**1**		**2**		**3**		**4**		**5**		**6**		**7**		**8**		**9**		**10**	
	***n***	***r***	***p***	***r***	***p***	***r***	***p***	***r***	***p***	***r***	***p***	***r***	***p***	***r***	***p***	***r***	***p***	***r***	***p***	***r***	***p***
1	891	−	−																		
2	891	0.561	0.000	−	−																
3	891	0.198	0.000	0.491	0.000	−	−														
4	891	0.359	0.000	0.656	0.000	0.592	0.000	−	−												
5	891	−0.290	0.000	−0.106	0.002	0.011	0.746	−0.023	0.492	−	−										
6	891	0.669	0.000	0.458	0.000	0.152	0.000	0.257	0.000	−0.247	0.000	−	−								
7	891	0.438	0.000	0.564	0.000	0.257	0.000	0.392	0.000	−0.061	0.070	0.505	0.000	−	−						
8	891	0.119	0.000	0.204	0.000	0.289	0.000	0.202	0.000	0.020	0.554	0.149	0.000	0.446	0.000	−	−				
9	891	0.272	0.000	0.365	0.000	0.239	0.000	0.397	0.000	−0.005	0.892	0.310	0.000	0.666	0.000	0.527	0.000	−	−		
10	891	−0.236	0.000	−0.142	0.000	−0.010	0.774	−0.037	0.274	0.684	0.000	−0.303	0.000	−0.111	0.001	−0.029	0.388	−0.028	0.400	−	−

*Source: Class climate, attitude climate study. The authors’ own computation. 1: W1 Anti-Roma prejudice. 2: W1 Acceptability of Roma prejudice by the respondent. 3: W1 Perception: teachers. 4: W1 Perception: classmates. 5: W1 Network diversity. 6: W2 Anti-Roma prejudice. 7: W2 Acceptability of Roma prejudice by the respondent. 8: W2 Perception: teachers. 9: W2 Perception: classmates. 10: W2 Network diversity.*

### Multilevel Models Analyzing the Factors Affecting Anti-Roma Prejudice

Given the nature of our data having students nested in school classes, we utilized multilevel linear regression models. We aimed to account for the effect of each factor affecting anti-Roma prejudice simultaneously; hence, we used multiple linear regression models. Based on the results of previous studies (presented in earlier chapters), a portion of the differences of prejudice among students may be attributable to the classes to which they go. To account for this, we fitted the multilevel models. We used Stata 13.0 and the xtmixed command with maximum likelihood option. In each model, we first tested whether there is a significant portion of class-level variance. In all our models, class-level variance proved to be significantly different from zero at the 5% level; thus, the use of multilevel models was justified. Class-level differences in mean prejudice may be attributable to contextual influences (class-level effects) or to individual-level differences in the composition of classes. As mentioned earlier, in these models, we examined the latter by adding individual-level predictors.

We first examined the factors affecting the level of anti-Roma prejudice in the two waves separately. Our independent variables were the following:

1. The respondent’s perception of the acceptability of anti-Roma prejudice among the respondent’s teachers.
2. The respondent’s perception of the acceptability of anti-Roma prejudice among the respondent’s classmates.
3. The diversity of the respondent’s network.
4. Gender as a control variable.

All independent variables in the models except for gender were transformed to a 0–10 scale. The reason for our decision was to provide information on scientific significance, not only on statistical significance^5^. The dependent variable is also measured on this scale.

Tables 3, 4 show the results of the multilevel linear regression analyses for the first and the second wave, respectively. We built the models step by step, in each step introducing an additional independent variable to be able to grasp the interferences between the variables and to investigate to which extent individual- and class-level differences are explained by the variables added to the model. To decide whether a significant portion of the differences in prejudice among students may be attributable to the classes in which they study, we introduced an empty model (Model 0). This model does not include any covariates but aims at partitioning the total variance in prejudice into variance that occurs between classes and that between pupils.

**TABLE 3:** Multilevel linear regression model for anti-Roma prejudice at the beginning of the school year (first measurement point).

	**Model 0**	**Model 1**	**Model 2**	**Model 3**	**Model 4**
**Fixed effects**	**Unstandardized regression coefficient (95% confidence interval)**				

Intercept	5.71*** (5.45, 5.97)	6.19*** (5.86, 6.52)	5.67*** (5.31, 6.03)	4.74*** (4.35, 5.13)	5.74*** (5.33, 6.15)
Gender (0 = male, 1 = female)		−0.93*** (−1.33, −0.53)	−0.88*** (−1.27, −0.49)	−0.80*** (−1.17, −0.43)	−0.67*** (−1.02, −0.33)
Perception of the acceptability of anti-Roma prejudice by teachers			0.23*** (0.15, 0.32)	0.00 (−0.09, 0.10)	0.02 (−0.07, 0.11)
Perception of the acceptability of anti-Roma prejudice by classmates				0.33*** (0.26, 0.40)	0.33*** (0.26, 0.39)
Network diversity					−0.37 (−0.44 -0.29)

**Random effects**	**Variance (95% confidence interval)**				

Class-level variance (significance: likelihood ratio test variance = 0)	0.36** (0.14, 0.96)	0.37** (0.12, 0.98)	0.25* (0.07, 0.83)	0.13 (0.02, 0.72)	0.03 (0.00, 11.16)
Individual level variance	8.29 (7.51, 9.12)	8.07 (7.34, 8.88)	7.90 (7.18, 8.70)	7.24 (6.60, 7.95)	6.71 (6.10, 7.34)
Proportional change in variance (PCV) by the new model					
Between classes	Reference	−2.5%	32.9%	48.8%	79.2%
Between individuals	Reference	2.4%	2.5%	8.0%	7.6%
ICC	0.04 (0.01–0.10)	0.04 (0.02–0.10)	0.03 (0.01–0.09)	0.02 (0.00–0.08)	0.00 (0.00–0.27)
LR chi-square difference between current and previous model (df), significance: improvement in goodness of fit as compared to the previous model	Reference	20.78 (1)***	29.93 (1)***	82.87 (1)***	80.80 (1)***

*p < 0.05, **p < 0.01, ***p < 0.001. Source: Class climate, attitude climate study. The authors’ own computation.

**TABLE 4:** Multilevel linear regression model for anti-Roma prejudice at the end of the school year (second measurement point).

	**Model 0**	**Model 1**	**Model 2**	**Model 3**	**Model 4**
**Fixed effects**	**Unstandardized regression coefficient (95% confidence interval)**				

Intercept	5.44*** (5.16, 5.73)	5.93*** (5.58, 6.27)	5.53*** (5.15, 5.91)	4.70*** (4.29, 5.11)	5.96*** (5.50, 6.42)
Gender (0 = male, 1 = female)		−0.94*** (−1.33, −0.55)	−0.98*** (−1.37, −0.60)	−0.91*** (−1.28, −0.54)	−0.89*** (−1.24, −0.54)
Perception of the acceptability of anti-Roma prejudice by teachers			0.16*** (0.09, 0.23)	−0.02 (−0.10, 0.06)	−0.03 (−0.11, 0.05)
Perception of the acceptability of anti-Roma prejudice by classmates				0.30*** (0.22, 0.36)	0.29*** (−0.22, 0.36)
Network diversity					−0.34*** (−0.41 -0.27)

**Random effects**	**Variance (95% confidence interval)**				

Class-level variance (significance: likelihood ratio test variance = 0)	0.58*** (0.28, 1.17)	0.53*** (0.25, 1.10)	0.46*** (0.21, 1.00)	0.32*** (0.14, 0.79)	0.22** (0.08, 0.64)
Individual level variance	8.29 (6.97, 8.47)	7.51 (6.81, 8.29)	2.72 (2.59, 2.85)	6.92 (6.30, 7.62)	6.35 (5.76, 7.02)
Proportional change in variance (PCV) by the new model					
Between classes	Reference	7.4%	13.3%	28.4%	31.6%
Between individuals	Reference	2.3%	1.7%	6.2%	8.6%
ICC	0.07 (0.03–0.13)	0.04 (0.02–0.10)	0.06 (0.03–0.12)	0.05 (0.02–0.10)	0.03 (0.01–0.09)
LR chi-square difference between current and previous model (df), significance: improvement in goodness of fit as compared to the previous model	Reference	22.18 (1)***	18.26 (1)***	63.58 (1)***	85.48 (1)***

*p < 0.05, **p < 0.01, ***p < 0.001. Source: Class climate, attitude climate study. The authors’ own computation.

Then, we started to build the models using the covariates starting with gender as we wanted to control for this variable in every model (Model 1). To test whether the effect of the perception of the teachers was at least partly mediated by the effect of the perception of the classmates, first we introduced the perception variable about the respondent’s teachers (Model 2) and then that of the classmates (Model 3). Finally, we included the variable measuring network diversity (Model 4). As the models are nested, we tested the improvement of the models’ goodness of fit using a likelihood ratio chi-square difference test in each step. For both waves, it can be said that in each step, the addition of a new explanatory variable significantly improved the fit of the model.

#### Individual-Level Predictors Affecting Anti-Roma Prejudice

In the empty model for the first wave, the intraclass correlation (ICC) is relatively small (4%). Since variance is always positive, the lower bound of the confidence interval cannot be negative. Therefore, it cannot include zero; thus, it cannot be used to infer about its significance. Instead, we used a likelihood ratio chi-square test to test whether the class-level variance significantly differs from zero. We found that the class-level variance significantly differs from zero, which can be interpreted as the justification for multilevel analysis. On these grounds, we might conclude that there is some evidence for a possible class-level contextual phenomenon shaping the difference in prejudice level. Alternatively, this clustering might be attributable to the different composition of classes (compositional *versus* contextual effect). As mentioned earlier, at this stage of the analysis, we examine the effect of individual-level variables. It is important to underline, that at the first time point, at the beginning of the first school year, ICC was found to be rather small, showing that only a small portion of differences in prejudice among students is attributable to their classes.

In the first wave, 93% of the class-level differences could be explained by the included individual-level predictors. It means that, in this wave, the compositions of classes according to gender, the perception of teachers and classmates, as well as network diversity could almost fully explain class-level differences^6^.

In the empty model for the second wave, the ICC is significant and bigger than in the first wave (7%), which means that class-level differences somewhat increased. When gender was included in the model (Model 1), the class-level residual variance went down by 7.4 percentage points (as compared to a 2.5 increase in Wave1^7^). It means that in this wave, class-level differences can be attributed more to the gender composition of the classes than in the first wave. However, in the second wave, only 60% of the class-level differences could be explained by the included individual-level predictors. It means that there are factors that affect class-level differences, which we have not considered yet. These factors might be other individual-level predictors but also contextual effects that could be captured by class-level covariates.

As for the nature of the relationships between the variables, very similar trends can be observed in both waves. Girls are less prejudiced against the Roma than boys, even if all the other independent variables are controlled for. The respondents who perceive their teachers or classmates more prejudiced are more prejudiced themselves. However, this effect is not significant anymore in the teachers’ case, when controlled for the effect of the perception of the classmates. In our research, it is impossible to decide whether the perception of the teachers affects that of the classmates or *vice versa*. In the first case, we can say that the effect of the perception of the teachers is fully mediated by the effect of the perception of the classmates (as predicted by Hypothesis 1c). It would mean that those who perceive their teachers more prejudiced perceive their classmates also more prejudiced, and this affects their own prejudice against the Roma. This does not only underline the importance of the classmates and how their attitudes are perceived in the formation of prejudice but also holds valuable information for understanding the role of teachers. This result would clearly suggest that teachers’ mere condemnation of prejudice would not be sufficient to counter the spread of prejudice, as students firstly follow the norms perceived among their classmates. However, we have to take into account also the second case if the perception of classmates has an effect on that of the teachers. It would mean that the perception of classmates has a strong (direct) positive effect on prejudice. Being positively correlated with perception of classmates, it creates a spurious relation between the latter and prejudice if it is not controlled for.

Our finding is in line with the Contact Hypothesis (as predicted by Hypothesis 2a): those having more diverse networks are less prejudiced against the Roma. Among the included predictors, network diversity has the strongest effect on anti-Roma prejudice. However, the perception of classmates is also an important determining factor. Effect sizes in the final models are more or less the same in the two waves. The only slight exception is that the difference between boys and girls after controlling for the other independent variables in the model is somewhat bigger in the second wave.

#### Change in Anti-Roma Prejudice Between the Two Waves

In the next step of our analysis, we aimed to grasp change in students’ anti-Roma prejudice. Table 5 shows our results. As before, all independent variables with the exception of gender were transformed to have a scale of 0–10. Note that the dependent variable is not transformed to 0–10, as it is measured on an interpretable scale: it measures the magnitude of change in the 0–10 scale prejudice. ICC in the empty model (Model 0) was around only 3% but significant.

**TABLE 5:** Multilevel linear regression model for the change of anti-Roma prejudice between the beginning and the end of the school year.

	**Model 0**	**Model 1**	**Model 2**
**Fixed effects**	**Unstandardized regression coefficient (95% confidence interval)**		

Intercept	−0.28 (−0.48, −0.08)**	−0.02 (−0.41, 0.37)	0.12 (−0.32, 0.55)
Gender (0 = male, 1 = female)		−0.07 (−0.39, 0.25)	−0.12 (−0.42, 0.19)
W1: Perception of the acceptability of anti-Roma prejudice by teachers		0.02 (−0.06, 0.10)	0.01 (−0.08, 0.11)
W1: Perception of the acceptability of anti-Roma prejudice by classmates		−0.11 (−0.17, −0.05)***	−0.06 (−0.14, 0.01)
W1: Network diversity		−0.02 (−0.41, 0.37)	0.01 (−0.07, 0.08)
Change in the perception of the acceptability of anti-Roma prejudice by teachers			0.00 (−0.07, 0.08)
Change in the perception of the acceptability of anti-Roma prejudice by classmates			0.08 (0.02, 0.14)*
Change in network diversity			−0.21 (−0.30, −0.12)***

**Random effects**	**Variance (95% confidence interval)**		

Class-level variance (significance: likelihood ratio test variance = 0)	0.58 (0.28, 1.17)**	0.17 (0.05, 0.55)*	0.11 (0.02, 0.51)
Individual level variance	5.41 (4.92, 5.95)	5.30 (4.82, 5.83)	5.16 (4.69, 5.68)
Proportional change in variance (PCV) by the new model			
Between classes	Reference	7.9%	37.8%
Between individuals	Reference	2.0%	2.6%
ICC	0.03 (0.01–0.09)	0.03 (0.01–0.09)	0.02 (0.00–0.08)
LR chi-square difference between current and previous model (df), significance: improvement in goodness of fit as compared to the previous model	Reference	18.98 (4)***	49.59 (7)***

*p < 0.05, **p < 0.01, ***p < 0.001. Source: Class climate, attitude climate study. The authors’ own computation.

In the first model (Model 1), we included the variables used in the previous models for the first time point. We found that although the improvement of the model fit as compared to the empty model is significant, there is only a single predictor that has a significant effect and that is the perception of the classmates at the beginning of the school year. Effect size is quite small and only 8% of class-level variance is attributable to the independent variables in the model. The coefficient is negative, which means that the more the respondent found his or her classmates dismissive against the Roma *already at the beginning of the school year*, the less his or her prejudice increased by the end of the school year. However, with the final interpretation of the coefficient, it is worth taking into account not only the beginning of the school year, but also the change between the end and the beginning of the year, that is, it is worth waiting for the next model, when, of course, except for gender, we also include the change of our covariates in the model.

In the next step, as mentioned above, apart from gender, we defined the change between the two waves in case off all explanatory variables. In the next model (Model 2), we entered these variables in addition to the already included ones. There were two significant variables in the model: the change in the perception of classmates and that of network diversity with the latter being much stronger. Based on the coefficients, it can be said that the more the perceived prejudice increased, the more his/her prejudice increased, and the more the network diversity increased, the less his/her prejudice increased by the end of the first year of secondary school. Returning to the perception of classmates, we see that the variable is not significant when we include the change of perception, which has a positive coefficient. This presumably can be explained by the fact that perception could decrease the most in those classes where the value of this variable was high at the beginning of the school year. However, it should be emphasized that the magnitude of the effect is very small. Based on our results concerning network diversity, we accepted Hypothesis 2b since we found that an increase in the diversity of the social network of the individual is correlated with a decline in the level of anti-Roma prejudice from the beginning to the end of the school year. Overall, 43% of the class-level differences were captured using the final model. At the same time, the differences in the change of anti-Roma prejudice at the individual level were explained only in a small part (about 5%) with the variables we included in our model; thus, further investigations are needed.

### Scrutinizing the Effect of Network Diversity in Greater Detail

We hypothesized that the diversity of the network, be it friends or acquaintances from any ethnic group, would reduce anti-Roma prejudices. Nevertheless, we considered it important to examine whether this assumption of ours holds true or whether it is in fact only Roma contacts that affect the prejudices against them. Moreover, our data are suitable to differentiate between direct and indirect contact effects. Direct contact measures whether the respondent him- or herself has Roma friends or acquaintances. While indirect contact measures whether the respondent has a friend or friends with Roma contacts. Like the other predictors, both of these variables were transformed to the 0–10 scale.

As the next step of our analysis, we examined the effect Roma network has on anti-Roma prejudices for both waves. We started from the last, largest model discussed in Section “Individual-Level Predictors Affecting Anti-Roma Prejudice” and varied it. As this model showed a non-significant class-level variance with a negligible ICC for wave 1, and the ICC was very small for wave 2, too, we decided to turn to single-level (ordinary) linear regression modeling. The results are shown in Table 6 for the first and in Table 7 for the second wave. To better understand the relationships between the variables, we built a series of models. In Model 1, we included those variables we used in the previous multilevel models. In the next model (Model 2), we changed the variable measuring network diversity that included all five outgroups to a variable measuring only Roma network, including both direct and indirect contacts. In Model 3, we included variables measuring both the overall and the Roma network. With this model, we sought to answer the question of what happens if we filter out the impact of the Roma network from the overall network, whether the latter still has its own effect. Finally, in the last model (Model 4), we included only the Roma network, but by breaking it down into direct and indirect contacts.

**TABLE 6:** Linear regression model to scrutinize the effect of network in-depth at the beginning of the school year (first measurement point).

	**Unstandardized regression coefficient (95% confidence interval)**			
	
	**Model 1**	**Model 2**	**Model 3**	**Model 4**
Intercept	5.74 (5.32, 6.15)***	5.82 (5.45, 6.20)***	5.89 (5.49, 6.29)***	5.81 (5.44, 6.19)***
Gender (0 = male, 1 = female)	−0.67 (−1.00, −0.32)***	−0.76 (−1.09, −0.43)***	−0.75 (−1.08, −0.42)***	−0.77 (−1.10, −0.44)***
Perception of the acceptability of anti-Roma prejudice by teachers	0.02 (−0.06, 0.11)	0.04 (−0.04, 0.13)	0.04 (−0.04, 0.13)	0.05 (−0.04, 0.13)
Perception of the acceptability of anti-Roma prejudice by classmates	0.33 (0.26, 0.39)***	0.30 (0.23, 0.36)***	0.30 (0.23, 0.36)***	0.29 (0.23, 0.36)***
Network diversity	−0.37 (−0.44, −0.29)***		−0.05 (−0.15, 0.05)	
Roma network		−0.32 (−0.37, 0.27)***	−0.30 (−0.36, −0.23)***	
Direct Roma network				−0.24 (−0.30, −0.18)***
Indirect Roma network				−0.08 (−0.13, −0.03)**
*p* (*F* test)	<0.001	<0.001	<0.001	<0.001
Adjusted *R*^2^	0.22	0.28	0.28	0.28

*p < 0.05, **p < 0.01, ***p < 0.001. Source: Class climate, attitude climate study. The authors’ own computation.

**TABLE 7:** Linear regression model to scrutinize the effect of network in-depth at the end of the school year (second measurement point).

	**Unstandardized regression coefficient (95% confidence interval)**			
	
	**Model 1**	**Model 2**	**Model 3**	**Model 4**
Intercept	5.90 (5.47, 6.35)***	6.14 (5.73, 6.55)***	6.21 (5.78, 6.64)***	6.14 (5.73, 6.54)***
Gender (0 = male, 1 = female)	−0.87 (−1.21, −0.54)***	−0.99 (−1.31, −0.66)***	−0.98 (−1.30, −0.65)***	−0.99 (−1.32, −0.67)***
Perception of the acceptability of anti-Roma prejudice by teachers	−0.02 (−0.10, 0.06)	−0.01 (−0.09, 0.07)	−0.01 (−0.09, 0.06)	−0.01 (−0.09, 0.07)
Perception of the acceptability of anti-Roma prejudice by classmates	0.30 (0.23, 0.36)***	0.25 (0.18, 0.31)***	0.25 (0.19, 0.32)***	0.25 (0.18, 0.31)***
Network diversity	−0.35 (−0.42, −0.28)***		−0.05 (−0.15, 0.04)	
Roma network		−0.31 (−0.36, −0.27)***	−0.29 (−0.36, −0.22)***	
Direct Roma network				−0.22 (−0.28, −0.16)***
Indirect Roma network				−0.10 (−0.15, −0.05)***
*p* (*F* test)	0.000	0.000	0.000	0.000
Adjusted *R*^2^	0.22	0.26	0.26	0.25

*p < 0.05, **p < 0.01, ***p < 0.001. Source: Class climate, attitude climate study. The authors’ own computation.

The analysis resulted in very similar results for both waves. When the variable measuring overall network diversity was replaced by the Roma network variable, we saw that the effect of the latter was not much weaker than that of the former. This has already suggested the results: when the two types of network variables were included together in the models, we found that if the effect of the Roma network is filtered out from the overall network, the latter loses its significant explanatory power; thus, we did not find secondary transfer effect of contact in relation to anti-Roma prejudice and rejected Hypothesis 2c. This might be due to the fact that prejudice against Roma is so deep-rooted and widespread in Hungary that only contact with members of this group is effective enough in reducing it. Separating direct and indirect Roma contacts—not surprisingly—we found that the former has a much stronger effect as predicted by Hypothesis 2d. However, it is important to note that even knowing someone who has Roma contacts can significantly reduce anti-Roma prejudice, after controlling for the number of direct Roma contacts. This result supported our Hypothesis 2e. These findings, pointing to the significance of contact, are especially important in light of the fact that in Hungary, schools are usually not integrated, with Roma students studying in either separate schools or separate classes (European Union Agency for Fundamental Rights, 2019).

### Perceived Non-acceptance of the Expression of Prejudice in the Classes

In our final analysis, we wanted to explore how perceptions of the acceptability of prejudiced expressions related to the change of the attitude climate during the first year of secondary school at the level of the classes. For this type of analysis, it was important to have data from a large proportion of respondents in each class. Therefore, for this analysis, we worked with a reduced subsample having selected only those classes in which the response rate was at least 66% in both rounds of data collection. Based on these selection criteria, our subsample consisted of 490 students from 21 classes from 15 secondary schools with a mean of 25 completed questionnaires per class. Students were 15 years of age on average (SD = 0.48) during the first wave of data collection. Among the respondents, 57% were female and all of them belonged to the majority ethnic group, Hungarian^8^.

For the analysis, we used three characteristics of each class, and in each case, we defined disagreement as 0–3 points on the 0- to 10-point scale:

1. How many percentage of the students in the class would not agree with anti-Roma remarks at the beginning of the school year?
2. How many percentage of the students in the class believe that most of their classmates would not agree with anti-Roma remarks at the beginning of the school year?
3. How many percentage of the students in the class would not agree with anti-Roma remarks at the end of the school year?

**FIGURE 1:**
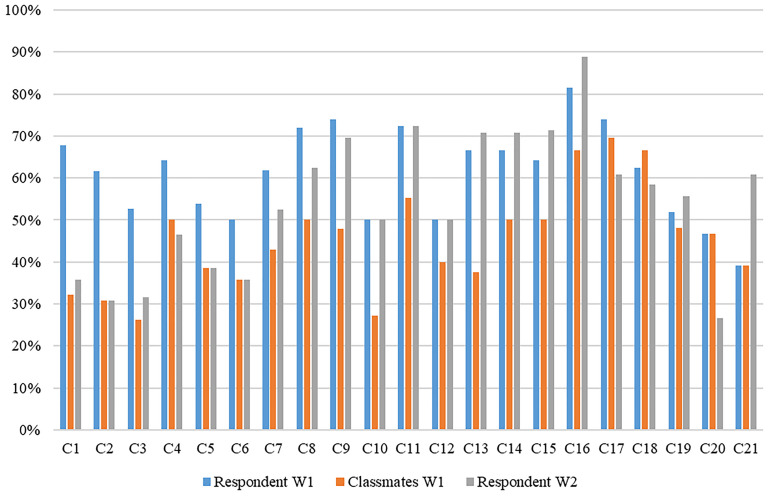
Class characteristics. Source: Class climate, attitude climate study. The authors’ own computation.

The first and the last variable measured the actual climate of the classes, while the second showed how this climate was perceived at the beginning of the school year (Figure 1). We found systematic (conservative) bias in 16 out of the 21 classes (C1–C16). In these classes, majority (at least 50%) of the respondents said at the beginning of the school year that they do not agree with anti-Roma remarks. However, they thought the same about their classmates to a much lesser extent, meaning that the difference was at least 10 percentage points. It means that, as expected (Hypothesis 3a), we found substantial pluralistic ignorance, with a systematic, conservative bias in about three-quarters of the participating classes, meaning that students underestimated their classmates’ non-acceptance of anti-Roma remarks.

Next, we examined what happened in these classes by the end of the school year. In half of these classes (C1–C8), the proportion of those who do not agree with anti-Roma remarks considerably decreased. In four cases, the difference was between 9 and 15 percentage points (C5–C8), and in two cases, it even exceeded 30 percentage points (C1–C2). As predicted by Hypothesis 3b, in line with the Theory of the Spiral of Silence (Noelle-Neumann, 1984) and previous studies of perceived norms and prejudice (for an overview, see Tankard and Paluck, 2016), in many cases, students, by the end of the year, have adjusted their own level of acceptance of prejudice to the falsely perceived class norm. Thus, in classes with conservative bias, less students have reported to reject anti-Roma remarks by the end of the year. Accordingly, in many cases, the misperceived norm became a reality, and students who, when entering a new class, would not have accepted anti-Roma remarks but falsely perceived their classmates of having a different opinion to theirs have reported higher acceptance of anti-Roma remarks at the end of the year, leading to a shift in majority view in some of the classes.

## Summary

The present study systematically mapped anti-Roma prejudice at the beginning and at the end of the first year of secondary school among Hungarian teenagers. By doing so, we aimed to recreate the context of school classes in which attitudes are formed and found that attitudes were adjusted to the perceived norms, most importantly to those of the classmates. Furthermore, we found that an increase in the number of contacts with Roma is coupled with a decrease in prejudice against them and that while close, direct contact with Roma has the strongest effect, even indirect contact was found to be coupled with significantly less prejudice against Roma people.

Though the mean level of anti-Roma prejudice has not changed substantially during the first year of secondary school, some individuals’ prejudice has decreased, while others’ have increased. At the same time, our findings suggest that school classes as normative contexts played a more important role for individual prejudice by the end of the school year. Similarly, the change in the perception of classmates’ acceptance of prejudice was found to be positively correlated with the change in the level of individual anti-Roma prejudice.

In order to better understand the interplay between perceived norms and the acceptance of prejudice in school classes, we also investigated how well students are able to sense the acceptability of prejudiced remarks at the beginning of the year. We found that students, at the beginning of the first year of secondary school, often underestimated their classmates’ non-acceptance of anti-Roma remarks. Consequently, we could see that, while at the beginning of the year, in many classes, students who would not have accepted anti-Roma remarks were in majority, this was not anymore the case by the end of the year. These findings suggest that students might have adjusted their attitudes to the falsely perceived class norm by stating less objection against anti-Roma remarks. These findings clearly underline the importance of perceived norms among classmates.

### Limitations and Future Research

Our study has a number of limitations that need to be addressed. First of all, we only surveyed students from secondary schools in Budapest; therefore, we cannot make any generalizations for the whole of Hungary or beyond. At the same time, given the comparability of our results to those of earlier studies and the fact that our results were in line with our theory-based hypotheses, there is no reason to exclude the possibility that similar patterns could be found elsewhere. A further limitation to our study is the ethnic homogeneity of our sample. It is very important to note that all students included in our study reported to belong to the majority ethnic group. While it is valuable to study intergroup phenomena in ethnically homogeneous settings, it is important to acknowledge that research including multi-ethnic classrooms would probably yield different and valuable results, definitely a path for further research.

Regarding our findings about the relationship between intergroup contact and prejudice, it is important to underline that our measure of contact was based on self-reports. Due to the high-level school segregation and the various problems related to the collection of ethnic data, it was not possible to include more reliable, objective measures of the dynamics of contacts in school classes. A study looking at intergroup friendships among Roma and non-Roma Hungarians would bring more clarity to this issue.

In our study, we mostly report correlational analyses, meaning that our findings have limited value for causal inferences. At the same time, the two-wave panel data allow comparisons between two time points and, therefore, our identification of correlates of attitude change has a stronger explanatory power than in the case of cross-sectional data. A similar study with at least three waves of data collection would allow further analyses, directly addressing questions of causality (Orth et al., 2020).

Finally, the quantitative nature of our study has its own limitations as it can only capture snapshots of the realities of adolescents, those at the time of the data collections. This type of data does not account for the events shaping the normative climates of the classes and the attitudes of the individuals in the 8 months between the two data collections. It is important to acknowledge that more in-depth qualitative research allowing a closer look at the everyday experiences of the students would enrich our understanding of the formation of attitudes in secondary school classes.

## Conclusion

The results of our current study can be placed within the field of research on prejudice in adolescence and the role of perceived norms and intergroup contact in shaping attitudes. Our study was conducted in Hungary, where social norms do not clearly proscribe ethnic prejudice, nor do schools tackle this problem. Our findings underline the importance of extending the scope of studies of prejudice to such contexts, for example, to the post-socialist countries of East-Central Europe, to better understand the role of social norms. Our study also draws attention to the problem of the acceptability of prejudice against the Roma, a grave social problem not only present in Hungary but plaguing the whole of Europe greatly (Zick et al., 2011; Kende et al., 2017).

Understanding the importance of the school class as the primary context of the formation of attitudes in adolescence has important implications for theory and practice alike. First of all, our findings underline the notion of prejudice being a social phenomenon, not only in the sense that it is directed toward others but also that it is produced by and learned in groups. Our study also brings new insights into our knowledge about intergroup contact in new settings, drawing attention to how making new outgroup contacts might serve as a shield against the perceived peer pressure of prejudice and give young people a chance to go beyond their prejudices. At the same time, our findings clearly demonstrate the significance of *perception* in the relationship between norms and prejudice. Our findings clearly show that adolescents, not having clear cues about the content of the class norm, tend to adjust to what they perceive, and what, in many cases, is not in line with their classmates’ actual views. This finding has clear implications for the practical work of prejudice reduction.

## What Does This Mean for Prejudice Reduction?

Piecing together the puzzle of perceived class norms and the formation of prejudice, our results show a clear path for prejudice reduction among adolescents. First of all, school classes should be considered as important terrain for such work, as they serve as the closest context of the formation of adolescents’ attitudes. The opportunity for social contact between the members of different groups in the context of a new school and a new class is of great importance as it may give a chance for the decline of prejudice. Finally, as students adjust their attitudes to what they perceive as accepted among their classmates, it is of utter importance to build class communities in which the social norms are in favor of equality and not of prejudice. This, however, is not sufficient in case the norms are not perceived correctly by the students in the class. Here, the role of the teacher might be of great importance as teachers might be able to foster the class norm of non-prejudice. This, however, should not be done by merely teaching it to the students but by ensuring that the Spiral of Silence cannot suppress the views of those who are not in favor of prejudice. Teachers, therefore, might do the most for the prevention of the spread of prejudice by creating an environment in which the voices of those who do not agree with prejudiced remarks are heard, ensuring that this becomes a viable and “visible” norm for students in the class.

We hope that our suggestions can initiate positive change and, consequently, to the question “*Whose norms, whose prejudice?*,” adolescents can answer, “*Our norms but without prejudice*.”

## Data Availability Statement

The datasets generated for this study are available on request to the corresponding author.

## Ethics Statement

The studies involving human participants were reviewed and approved by Central European University Ethics Review Board. Written informed consent to participate in this study was provided by the participants’ legal guardian/next of kin.

## Author Contributions

LV planned the study, collected and compiled the dataset, and drafted the manuscript. IB and RN planned the data analyses together with LV, built the models, and analyzed the data. IB wrote the results section and commented on the whole manuscript. All authors approved the submission of the final version of the manuscript.

## Conflict of Interest

The authors declare that the research was conducted in the absence of any commercial or financial relationships that could be construed as a potential conflict of interest.

## Footnotes

1 The study was approved by Central European University’s Ethical Research Committee.

2 The data set and further materials used for the present study are available to readers upon request from the corresponding author.

3 Before creating the composite index, we turned over the values of the original variables.

4 As we analyze the interrelations of all the variables mentioned above, we refrained from including the respondents’ opinion about a classmate’s anti-Roma remark into the composite index measuring anti-Roma prejudice.

5 The original variables had a similar scale, with a minimum value of 0, and the maximum values differed only for technical reasons (being *ad hoc* characteristics of the measurement tool). Therefore, to make the scales comparable, we divided the variables by the tenth of their maximum values. This way, the interpretation of effect sizes (e.g., regression coefficients) is straightforward; it is convenient to consider (causal) effect of changing the predictors, and we can make comparisons not only between predictors but between time points as well.

6 As mentioned earlier, we ran the models already in the first wave only for those who responded in both waves. Examining the impact of this dropout, we found that the model including all respondents has higher intraclass correlation: 10% compared to 4% after the dropout. It means that the omitted students and classes reduce class-level heterogeneity. However, it is important to note that the remaining heterogeneity is still significant. Without the dropout, approximately two-thirds of class-level variance was attributable to pupil-level covariates, which is lower than in the model we finally ran. However, examining the nature of the relationship between the variables, it is important to highlight that the direction, magnitude, and significance of the relationships are similar to those in the model for the subsample.

7 An increase in the residual class-level variance is mathematically possible under maximum likelihood estimation (Raudenbush and Bryk, 2002, p. 150) but as this is not the substantive focus of the present paper, we do not explore it further.

8 The mean value for anti-Roma prejudice in the first measurement point did not differ significantly for the sample used in the multilevel models and this reduced subsample. However, the variance of the latter group was significantly lower (*p* < 0.05). In the second measurement point, neither the mean nor the variance of the two groups differ.
